# Uncommon T12 Burst Fracture after an Epileptic Crisis

**DOI:** 10.1155/2011/726373

**Published:** 2011-09-22

**Authors:** Akiki Alian

**Affiliations:** Traumatology Department, Hopital du Chablais, 1870 Monthey, Switzerland

## Abstract

People having an epileptic crisis present to the hospital with an altered mental status and generalised fatigue. The most common orthopaedic pathology associated to epilepsy is the undiagnosed posterior shoulder dislocation. These same patients often complain from back pain that is often neglected and misdiagnosed as muscular contracture following the epilepsy crisis. We describe here the case of a patient who presented after here epilepsy crisis with back pain. Investigations revealed an uncommon burst fracture that needed a surgical treatment. *Conclusion*. Back pain after an epileptic crisis should be investigated more seriously with an adequate clinical examination and a minimum of a radiography of the back.

## 1. Introduction

Patients suffering from epilepsy have an increased risk of fractures. This can be associated with the trauma induced directly by the seizure itself or resulting from a fall due to the seizure. If posterior shoulder dislocation seems to be the most common clinical entity associated to epileptic crisis, vertebral fractures are commonly overlooked, because these fractures tend to be asymptomatic [1, 2]. We describe here a case of a “diabolo” burst fracture of T12 body, which occurred during a single incidence of grand mal seizure and was diagnosed only two days after the seizure episode due to the appearance of back pain.

## 2. Case Report

A-thirty-five year old female was admitted to our emergency unit with the main complaint of low back pain that appeared one day ago without any history of trauma. She only reported that two days ago she was admitted to another hospital after an epileptic crisis where she was treated and observed for 24 hours.

Anamnestically she has a history of intractable epilepsy diagnosed in her early 20 years. 

She admits an irregular consumption of her medications. Otherwise she is in good health.

On inspection, no signs of trauma or bruises were found on her back or buttocks. Percussion of her spinal column revealed pain in the midlumbar area accompanied by an important contracture of the paraspinal muscles. Neurological examination did not reveal any alteration in sensitivity of the upper extremities, lower extremities, or in the presacral area.

Deep tendon reflexes were normal. On rectal examination, the sphincteric tone was normal.

The patient was able to stand and walk. Urinalysis tests were normal as well as blood chemistry values.

**Figure 1:**
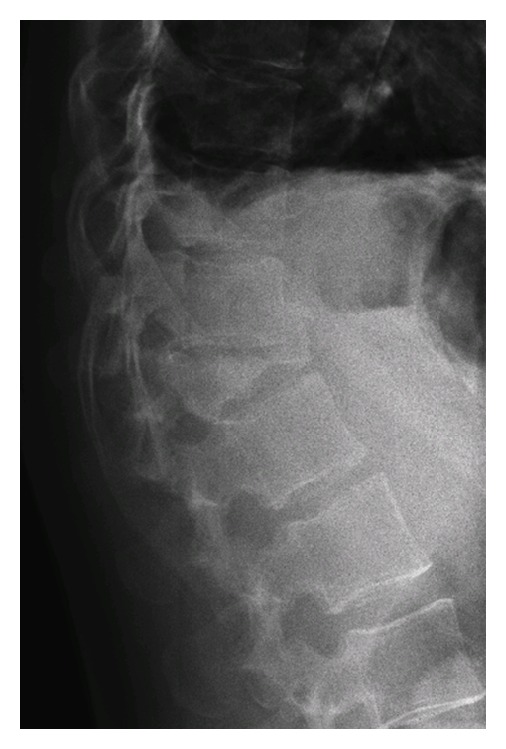
X-ray showing a burst fracture of T12.

**Figure 2:**
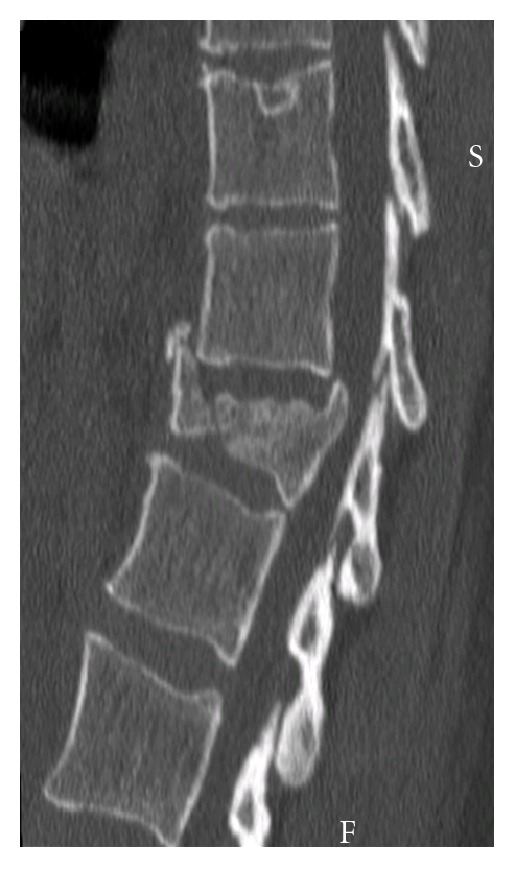
CT Scan confirming the burst fracture of T12.

Because of her symptomatology, thoracolumbar spine radiography was obtained. It showed a burst fracture of T12 with a local kyphosis of 35° (Figure 1). Investigations were completed by a CT-Scan of the lesion that confirmed the diagnosis of a “Diablo” burst fracture of T12 or Denis B type fracture, with disruption of the posterior wall but with no neurological compromise (Figure 2).

**Figure 3:**
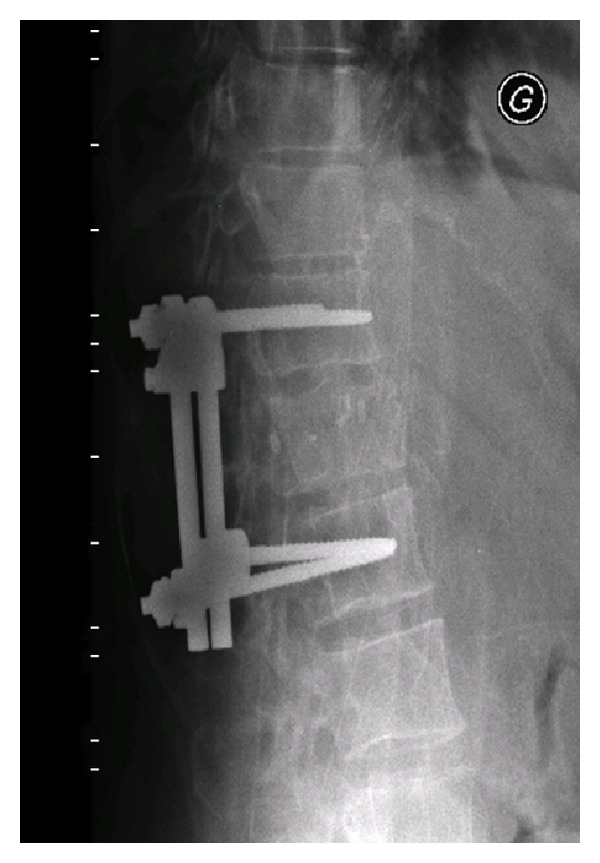
Postoperative lateral X-ray showing the height restitution of T12.

**Figure 4:**
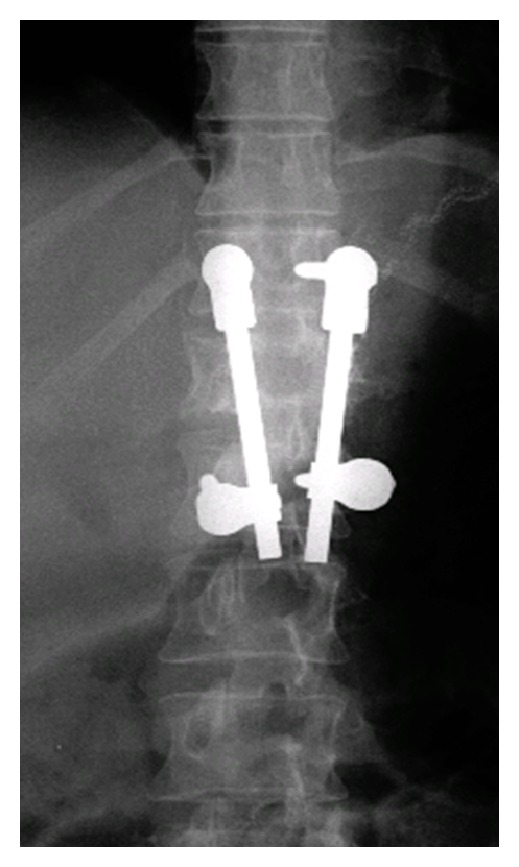
Postoperative AP showing view of a good reduction of the T12 fracture.

The patient was then admitted to the hospital where she underwent a 2-stage posteroanterior stabilisation and fixation of her column from T11 till L1. Postoperatively, she had no neurological lesions and resumed walking with crutches for the first 4 weeks (Figures 3 and 4).

## 3. Discussion

Epileptic seizures affect approximately 0.2–0.5% of the general population. Up to 3% of epileptic patients are injured by either direct or accidental consequence of epileptic seizures [1–4]. Seizure-related fracture varies in terms of location. While the spine is the most common epilepsy related fracture site, other potential locations include facial bones, glenohumeral joint, humerus, distal radius, and proximal humerus [1–3, 5]. Bilateral posterior dislocation or fracture dislocation of the shoulders is highly suggestive of seizure-related injuries [1, 4]. The incidence of symptomatic spinal fracture from a seizure is rare and is estimated to occur in 1% of epileptic patients, whereas asymptomatic spinal fracture may be as high as 15-16%. The most common fracture location in the vertebral column is the upper to midthoracic region (T3–T8), in contrast to thoracolumbar junction or the cervical spine fractures frequently found in patients with external trauma [6, 7]. Usually this distinctive distribution occurs because compressive forces during contraction of the muscles are concentrated along the anterior and middle columns of the midthoracic kyphosis curves [6, 7]. A burst fracture is a descriptive term for an injury to the spine in which the vertebral body is severely compressed. The term burst implies that the margins of the vertebral body spread out in all directions. This is a much more severe injury than a compression fracture for two reasons. With the bony margins spreading out in all directions the spinal cord is liable to be injured. The bony fragment that is spread out toward the spinal cord can bruise the spinal cord causing paralysis or partial neurological injury. Spinal burst fracture often occur at the thoracolumbar junction and accounts for about 15% of spinal injuries [8]. Denis classified burst fractures into five categories on the basis of radiographic appearance [9]. Type B is the most common and involves the superior end plate and retropulsion of the superoposterior cortex. Type C is rare and involves fracture of the inferior end plate only. Type A includes fracture of both end plates. Type D is a burst rotation injury while type D is a lateral flexion burst injury.

Neurological injury has been reported to occur in 30% ± 60% of the patients with thoracolumbar burst fractures [8, 9].

Limb et al. [10] suggest that the static image of the canal obtained by the computerized tomography scans hours or days after the injury does not necessarily reflect the displacement at the time of injury, which is what determines the initial neurological insult. The degree of spinal canal narrowing reflects the canal resting position of the vertebral body fragments after the trauma. This would explain why the present study failed to show any association between the extent of canal compromise and the extent of neurological deficit. In burst fractures of the thoracolumbar and lumbar spine, there is no correlation between the neurological deficit and the recovery pattern with the extent of canal compromise.

Burst fracture of vertebral bodies after a grand mal seizure is not common. T12 burst fracture as a direct consequence of an epileptic crisis, to best of our knowledge, has not been related in the literature. Probably this is attributed to the fact that the seizure trauma resulting from axial skeletal contraction force is usually not sufficient to cause burst fracture.

Antiepileptic drugs are known to increase the risk of fracture in epileptic patients through the reduction of the bone mineral density [11–13]. But so far, no evidence exists to suggest that spinal precautions are necessary in all seizure patients, and the routine use of spinal precautions in uncomplicated seizure patient's has been questioned [14, 15].

## 4. Conclusion

In conclusion, forceful contraction during a convulsive seizure can result in vertebral burst fracture, although this is not very common.

The absence of external trauma and the postictal consciousness disturbance usually delay the early diagnosis.

A complaint of back pain should be looked for in epileptic patients after a grand mal seizure, and if it is positive, it should raise a strong suspicion of vertebral fracture and thus should be evaluated radiographically.
